# ‘I forget to do pressure relief’: Personal factors influencing the prevention of secondary health conditions in people with spinal cord injury, South Africa

**DOI:** 10.4102/sajp.v77i1.1493

**Published:** 2021-03-15

**Authors:** Sonti Pilusa, Hellen Myezwa, Joanne Potterton

**Affiliations:** 1 Department of Physiotherapy, Faculty of Health Sciences, University of the Witwatersrand, Johannesburg, South Africa

**Keywords:** secondary health conditions, spinal cord injuries, prevention, factors, ICF

## Abstract

**Background:**

Across the lifespan, people with spinal cord injury (SCI) may experience preventable secondary health conditions (SHCs) such as pressures sores, muscle spasms and urinary tract infections (UTIs). Some factors influencing prevention of SHCs include social support, poor access to care and the prevention style of individuals. There is limited research on these factors.

**Objective:**

To explore personal factors influencing the prevention of SHCs in people with SCI.

**Method:**

An explorative qualitative study included participants recruited in an outpatient department at a rehabilitation hospital. Semi-structured interviews were conducted with patients with SCI. Interviews were transcribed verbatim. Data analysis was conducted using content analysis.

**Results:**

Seventeen individuals with SCI were interviewed. From the interview analysis, six personal factors were identified, namely, socio-economic status; mental well-being (forgetfulness, beliefs, attitude); lack of knowledge of SHCs and prevention; lifestyle choices and practising prevention care; patient activation (self-management, problem-solving, resilience, self-awareness, help-seeking behaviour) and owning an appropriate assistive device.

**Conclusion:**

Socio-economic status, mental well-being, knowledge of SHCs and prevention care, behaviour patterns, patient activation and owning an appropriate assistive device can influence prevention of SHCs. To enhance patient-oriented care, a model of care for people with SCI should consider these factors when developing prevention strategies. Future research could look into identifying environmental factors that influence the prevention of SHCs in people with SCI.

**Clinical implications:**

Tailored prevention strategies need to be developed, health professionals must ask patients about individual factors that may be barriers or facilitators to preventing secondary health conditions.

## Introduction

The sustainable development goal number 3 aims to ensure healthy lives and promote well-being for all including people with disabilities (United Nations 2018). Despite health promotion and preventive care for people with disabilities being important, they are often neglected (Lofters et al. 2019). Health promotion and preventative care are a necessity because people with disabilities are at a higher risk for developing preventable secondary complications commonly known as secondary health conditions (SHCs) (Adriaansen et al. 2013).

Secondary health conditions can occur during the lifespan of a person with a spinal cord injury (SCI), and are not caused by the primary injury (SCI) but are related to living with a disability (Rimmer, Chen & Hsieh 2011). They include pressure sores, pain, muscle spasms, bowel and bladder problems. Secondary health conditions can occur during the acute and rehabilitation phases (Wahman et al. 2019), and in the post-discharge phase (Adriaansen et al. 2016), negatively impacting health outcomes. Given that there is no cure for SCI, care should include the promotion of well-being and prevention of SHCs.

The presence of SHCs in the life of people with SCI can increase levels of disability (Callaway et al. 2015), decrease life expectancy (Oderud 2014) and increase hospital readmission rates (Mashola, Olorunju & Mothabeng 2019). Up to 80% of readmissions amongst people with SCI in one private rehabilitation hospital in South Africa were because of SHCs such as pressure sores and urinary tract infection (UTI) and patients were readmitted up to four times for these preventable SHCs (Mashola et al. 2019). It is still not clear what causes patients with SCI to be re-hospitalised several times for the same SHCs. What could be hindering the prevention of the SHC? Why do some patients manage to prevent SHCs and others do not? To improve health outcomes for people with SCI, we need to understand the factors influencing the prevention of SHCs.

To appreciate the degree of functioning, disability and to develop patient-specific interventions, it is important to understand patients’ personal factors. According to the International Classification of Functioning, Disability, and Health (ICF), individual (personal) factors are intrinsic characteristic which are not part of the health condition that can enhance or inhibit health and shape the experience of disability (Grotkamp et al. 2019; World Health Organization 2013). These factors include gender, race, age, presence of other health conditions, lifestyle, habits, coping styles, educational level, profession, past and current experience and psychological assets such as coping style and self-efficacy (Grotkamp et al. 2019; World Health Organization 2013). Considering factors at an individual level in patient assessments and when developing tailored interventions is good practice, promotes patient-centred care and is in line with the Convention on the Rights of Persons with Disabilities, Article 26 on the assessment for rehabilitation based on an individual’s strength and weakness (United Nations 2006).

Notwithstanding that preventative care is a collaborative effort amoung health professionals, caregivers and patients, the patient’s role and intrinsic influences in preventative care are important (Zanini et al. 2019, 2020).

Patients’ activation (Greene & Hibbard 2012); outlook and control over personal care (Munce et al. 2014); knowledge levels of SHCs; attitude to prevention care; belief in personal susceptibility and taking responsibility for personal well-being influence prevention care practice (King, Porter & Vertiz 2008; Zanini et al. 2020). Moreover, belief in one’s ability to self-manage has been identified as an essential component of managing a chronic condition (Disler, Gallagher & Davidson 2012; McCorkle et al. 2011).

Other factors that influence the prevention of SHCs that have been mentioned in the literature include participation in neuro-musculoskeletal health behaviours such as range of motion and muscle strengthening exercises (Mashola & Mothabeng 2019), access to assistive devices (Burns & O’Connell 2012; Oderud 2014) and lack of health education on SHCs prevention (Fuseini, Aniteye & Kofi-Helegbe 2018). Intrinsic factors that are seldom linked to the discussion on preventive care, but may explain why some are successful and others not despite doing the right thing, are factors at a molecular level. There is evidence linking the reoccurrence of UTI to malfunctioning of an innate immune activation gene (Godaly, Ambite & Svanborg 2015), meaning that even if an individual practices good bladder management UTI will still occur. Nutritional deficiencies have also been linked to delayed pressure sore healing (Bhagat et al. 2018). Thus, intrinsic factors influencing health, health outcomes and prevention of diseases are many, ranging from genetic predisposition to psychological, mental and physical status.

No study has comprehensively examined personal factors (intrinsic factors) that can influence the prevention of SHCs in people with SCI in South Africa. An understanding of the factors inhibiting or enhancing prevention of SHCs can help clinicians to develop targeted interventions that enhance knowledge, self-management skills, adoption of health-promoting behaviours and foster responsibility for personal well-being. Thus, our study explored and identified personal factors influencing the prevention of SHCs among people with SCI.

## Method

An exploratory qualitative study was used to explore the individual factors as described by the ICF (World Health Organization 2013) as the ICF can be used by health professionals to understand factors influencing health outcomes and to inform interventions.

Our study was undertaken at a public tertiary rehabilitation hospital in South Africa, which offers inpatient and outpatient rehabilitation services to patients with stroke, SCI, amputation and head injuries. The rehabilitation hospital is a 79-bed hospital that caters for patients from within and outside the province. The percentage of patients admitted with SCI fluctuates seasonally; after the festive season it is 60% of the admission and drops to 40% during the year.

Purposive sampling was used to recruit potential key informants with SCI attending the outpatient department. Only patients with a SCI diagnosis (traumatic or non-traumatic) and 18 years and above were recruited.

### Data collection

Semi-structured interviews were conducted by the first author using an interview guide in the language in which the key informant was familiar. The interview guide had two parts: demographic data and open-ended questions with probing questions on factors related to the prevention of SHCs. Demographic data included age, gender, educational levels and information on the injury. The participants were asked how they prevented SHCs and what made prevention easier or harder. A pilot study was conducted to test the feasibility of our study and to check for any changes that could be done to improve the clarity of questions and the process.

After informed consent and permission to audio-record the interviews were granted by each participant, interviews were conducted from July 2018 to October 2019. The interviews lasted approximately 60 min and data collection continued until data saturation was reached. The interviews were audio-recorded and debriefing sessions were conducted to discuss the research process, findings and data analysis with the co-authors (H.M. and J.P.).

### Data analysis

All the interviews were transcribed verbatim for data analysis. The analysis was conducted using content analysis, a continuous and reflective process of identifying meaning units, coding, grouping similar codes into categories and themes (Erlingsson & Brysiewicz 2017). Depending on the focus of a particular study, abstraction of data can be at a literal level or the manifest content which can then end at the categories level, answering the ‘what’ question (Graneheim & Lundman 2004). Alternatively, the analysis can go further into the latent content, where themes are identified to answer the ‘how’ question. The transcripts were read and reread to ensure accuracy of transcription and translation and to get an overall sense of the interviews. Inductive coding was conducted independently on one transcript by the authors and an external expert in qualitative data analysis and public health.

Thereafter separate discussions on the findings were conducted to compare the generated codes. In the discussion, similar codes were grouped together to form subcategories and categories. A preliminary coding framework was developed which was used to code the rest of the transcripts deductively by the first author. The focus of the analysis was on the description of the different factors at play at a personal level influencing the prevention of SHCs, thus the level of analysis ended at the category level. A manual display of the categories and subcategories was presented to the other authors which was followed by a discussion and renaming some of the subcategories and categories. MAXQDA (qualitative data analysis) version 2018.2 was used to manage and analyse data.

To ensure trustworthiness of the data, the following strategies were utilised (Nowell et al. 2017). Credibility was ensured by including only participants who met the inclusion criteria, and who could give a clear narration of the study phenomena. Secondly, regular meetings and debriefing sessions to discuss the research process, data collection, findings and data analysis were conducted with the co-authors. Investigator triangulation was conducted when developing the preliminary coding framework, which was subsequently used to code the rest of the transcripts providing a trail of the coding process (Graneheim & Lundman 2004). To ensure dependability, a detailed description of the data collection, analysis and interpretation was outlined, and a code–recode procedure was used – coding of a segment of data in two separate sessions. Field notes were kept during the data collection phase. For transferability, the context and the demographic data of the participants were explained in detail.

### Ethical considerations

Permission to use the study site for data collection was granted by the rehabilitation hospitals. Our study was approved by the Human Research Ethics Committee of the University of the Witwatersrand (M170938) and registered with the South African. National Health Research Database (GP201712036).

## Results

Seventeen patients with SCI were interviewed. Table 1 provides an overview of the demographic data of the participants. The majority of the participants with SCI were male and had traumatic SCI.

**TABLE 1:** Demographic profile of the participants (*n* = 17).

Participants with spinal cord injury	*N*	%
**Age in years**		
Mean (SD)	44.5	13.1
Range	27–72	-
**Gender, *n* (%)**		
Male	14	82.4
Female	3	17.6
**Employed, *n* (%)**		
Yes	5	29.4
No	12	70.6
**Education level**		
Tertiary education	4	23.5
Matric	5	29.4
High school	6	35.3
Primary school	2	11.8
**Time since injury**		
Mean (SD)	9	7.1
Range (years)	1–30	-
**Cause of injury, *n* (%)**		
Trauma	14	82.4
Non-trauma	3	17.6
**Type of spinal cord injury**		
Paraplegia	14	82.4
Quadriplegia	3	17.6
**Completeness of the injury**		
Incomplete	4	23.5
Complete	13	76.5
**Level of the injury**		
C1–C4	2	11.8
C5–T1	1	5.9
T2–T6	3	17.6
T7–T12	9	52.9
L1–L5	2	11.8
**Assistive device**		
Wheelchair	14	82.3
Walking aid	2	11.8
None	1	5.9

SD, standard deviation.

From the interview analysis, six categories were identified, namely, socio-economic status; mental well-being; knowledge on SHCs and prevention; behaviour patterns, patient activation and owning an appropriate assistive device. To protect anonymity, the quotes are numbered (Participant number, gender, age). The results are discussed below and summarised in Tables 2–6.

**TABLE 2:** Socio-economic status.

Subcategory	Quote
Finances not enough	‘I don’t work, I buy food with grant money and when the diapers get finished so it becomes hard, so often when I have diarrhoea, I don’t leave the house, I just sit and I use the linen saver that they gave us.’ (P12, male, 35 years old)
	‘The money we get is not enough to buy all these and for travelling purposes – sometimes we even miss our check-up dates because we run out of funds.’ (P1, male, 39 years old)
Access to finances	‘I feel I am very privileged to have a medical aid that pays Botox for my bladder.’ (P16, female, 61 years old)
	‘I actually took out the money I saved up to buy myself this cushion, since I moved to the air cushion, I haven’t had that problem anymore.’ (P11, male, 35 years old)

**TABLE 3:** Mental well-being.

Subcategory	Quote
Forgetfulness	‘I used to forget to do pressure relief.’ (P4, male, 32 years old)
Beliefs	‘You only get to believe this after you have developed pressure sores – seeing that what you were told is actually true.’ (P1, male, 39 years old)
	‘I did not listen. I never thought pressure sores will happen to me that easily …. I proved it … it happened and this is the second time it happens.’ (P14, male, 34 years old)
Feelings	‘What gets on our way sometimes is the shame we have about our conditions … we don’t want to show our friends the kinds of things we have to do in such conditions.’ (P1, male, 39 years old)
	‘[*Reason for not doing pressure reliefs*] remember I told I was abusing drugs that time? I was on drugs and I just didn’t care. You see, when you live with an injury, you get angry and sometimes you do just wish to die.’ (P3, male, 44 years old)
Attitude	‘I learnt the hard way. I got pressure sores, usually, the thing is you underestimate the pressure sore. You think it will heal, no way.’ (P15, male, 50 years old)
	‘If I want to drink a lot of brandy and coke the whole weekend, I know Monday I’m going to suffer with my bladder. … So, Monday I’ll deal with it.’ (P17, male, 36 years old)
Past experience	‘I also took it very light but I ended up seeing it myself no one can tell me I have experience that this thing is dangerous it can kill you in a month kill you.’ (P14, male, 34 years old)
	‘The doctor once prescribed some pills for me and they didn’t work, he prescribed more and they didn’t work so I gave up on trying.’ (P12, male, 35 years old)

**TABLE 4:** Knowledge on secondary health conditions and prevention.

Subcategory	Quote
Lack of knowledge on SHC	‘I didn’t know how a pressure sore looks like when it develops. I didn’t understand, I just thought it’s a sore and it will be fine.’ (P15, male, 50 years old)
	‘I really do not understand. You can lift and relief and do all sorts of things, but I don’t think it makes any difference it is the same.’ (P3, male, 44 years old)
Lack of knowledge on prevention of SHCs	‘The cushion I was using was old, since I was discharged with it in 2017; I didn’t know that a cushion gets changed every 6 months.’ (P15, male, 50 years old)

SHCs, secondary health conditions.

**TABLE 5:** Behaviour patterns.

Subcategory	Quote
Lifestyle choices	‘What helps me with pain management is a stimulating activity, or if I exercise.’ (P16, female, 61 years old)
	‘For me I think my pressure sores were caused by the drug [*nyaope, “ohock”, tik*]. When you take drugs you relax just sit still.’ (P3, male, 44 years old)
	‘The food we eat at home is not good for us for your condition … They recommend that we eat a lot of fruit and veggies … but we don’t eat those foods, we eat pap instead.’ (P14, male, 34 years old)
Prevention care practice	‘I didn’t treat the pressure sore according to how they had told me I started sitting on the wheelchair which I wasn’t supposed to.’ (P14, male, 34 years old)
	‘I do a lot of pressure reliefs.’ (P12, male, 35 years old)
	‘Even now – when I get healed, I have to continue doing it [*pressure relief*], otherwise the bed sores will come back or I will die.’ (P3, male, 44 years old

**TABLE 6:** Patient activation.

Subcategory	Quote
Self-management	‘I get up just after 5 am in the morning, then I take a shower, and do coloplasting every day and after the shower I get into the bed I use methylated spirits for my buttock. I can feel with my hands that my skin is actually feeling good.’ (P10, male, 66 years old)
Problem-solving	‘I am paralyzed, I have to get in the bath, I’ve burnt my feet once. So, what am I going to do differently? Change the showerhead, do this, do that. I mean, that’s something you’ve got to figure out yourself.’ (P17, male, 36 years old)
Resilience	‘I fell out of my wheelchair and I learnt a valuable lesson that day – you must always keep your cell phone with you if you are disabled … I did not have my phone with me. I lay on the floor for over three hours.’ (P16, female, 61 years old)
Self-awareness	‘Like when I have pain in my left foot, I would sweat on my right side. So then you know you have to check for something that’s wrong.’ (P15, male, 50 years old)
	‘I must be more aware of the body, what the body needs, you know … when I’m feeling tired I don’t want to eat … I force myself to eat something.’ (P12, male, 35 years old)
Help-seeking behaviour	‘I have never had pressure sores because when I bath I ask my girlfriend to check my skin.’ (P12, male, 35 years old)
	‘Will only mention the bladder leakage to the doctor only when it gets worse.’ (P9, male, 40 years old)
Seeking help late	‘What could have made it easy for me is upon seeing the bedsore for the first time, I should have went and gotten things to dress it same time.’ (P14, male, 34 years old)

### Socio-economic status of patients with spinal cord injury

Many participants highlighted economic challenges. Participants stated that a lack of adequate finances leads them to not being able to buy healthy food, unable to buy medication or being able to access healthcare services.

High economic status helped patients afford care and assistive devices, thus was perceived as a facilitator for the prevention of SHCs. The subcategories and quotes are illustrated in Table 2.

### Mental health status on patients with spinal cord injury

The analysis further showed how mental well-being can influence the prevention of SHCs. The subcategories under mental well-being include forgetfulness, beliefs, feeling and attitudes towards prevention of SHCs and past experience (Table 3).

### Knowledge on secondary health conditions and prevention

Some of the participants lacked knowledge on SHCs and how to prevent their occurrence. Quotes for knowledge on SHCs and prevention care are summarised in Table 4.

### Behaviour patterns

There were behaviour patterns that facilitate prevention of SHCs such as health promoting lifestyle choices (exercising, quitting smoking and drinking alcohol). On the contrary, some patients even after being educated were not compliant with preventative care practice for SHCs. Some of the participants, although they knew about prevention of SHCs, were not compliant in preventing their occurrence. Subcategories and quotes are illustrated in Table 5.

### Patient activation

To prevent and manage SHCs, participants practised self-management, problem-solving, resilience and self-awareness, and help-seeking behaviour. Some participants delayed seeking help early, thus negatively affecting their health outcomes. Subcategories and quotes for activated patients are illustrated in Table 6.

### Owning an appropriate assistive device

Owning an appropriate assistive device was highlighted as playing an important role in the prevention of SHCs, for example, the use of a wheelchair cushion and special mattress to prevent SHCs. Patients who owned special assistive devices were the ones who could afford to buy one or had received one as a donation:

‘I have that special mattress. If it wasn’t for that mattress, I would have had many pressure sores.’ (P15, male, 50 years old)

‘But the major thing I feel also for me not getting pressure sores being in a chair is the cushion I’m sitting on is one of those air cushions, the Roho cushions, ‘cos you don’t need to do that constant pressure release.’ (P11, male, 35 years old)

## Discussion

**FIGURE 1:**
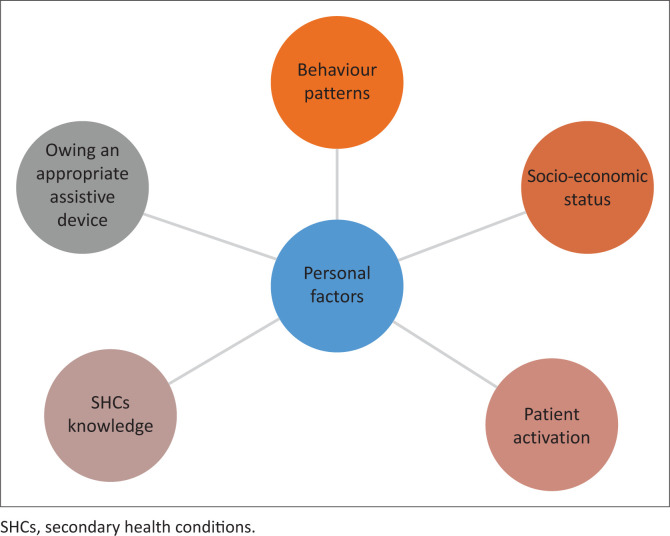
Personal factors influencing the prevention of secondary health conditions.

Our study identified personal factors that may influence the prevention of SHCs. The identified personal factors are socio-economic status, mental well-being, knowledge on SHCs and prevention; behaviour patterns, patient activation and owning an appropriate assistive device, as shown in Figure 1.

The socio-economic status of an individual can affect health outcomes and prevention of SHCs. The limitations because of impairments can affect participation in employment resulting in economic vulnerability. The economic vulnerability can also be from the high cost of living with disability-related extra costs for assistive devices, caregiver payment, hiring alternative transport (Hanass-Hancock et al. 2017) and social disability grants being used to cover household needs leaving little for the needs of a person with a disability (Kelly 2019). This may lead to pressure sore development, for example, because of the inability to buy assistive devices (Oderud 2014).

Patients with high incomes could afford to buy specialised assistive devices such as wheelchair cushions to aid in preventing SHCs. Those with low income could not even manage to get a healthy diet compromising their nutritional status, thus increasing the risk of the development of SHCs and delaying wound healing for those with an existing pressure sore (Bhagat et al. 2018). There is also a link between lack of finances and mental problems (Zürcher, Tough & Fekete 2019).

Employment is a means of securing an improved economic status, with the evidence indicating that participation in the labour market is generally low for people with disabilities, and the opportunity to gain employment is worsened by low education levels (Hanass-Hancock & McKenzie 2017). In our study, only five participants with SCI were employed, indicating a need for vocational rehabilitation to increase employability. Therefore, as part of vocational rehabilitation, opportunities to learn new skills and to study further should be explored for patients with low education levels. Also, government departments must include people with disabilities in key developmental programmes such as the Expanded Public Works Programme, Sector Education Training Authorities and business training opportunities. Initiatives like this could facilitate the participation of people with SCI in the open market to decrease economic vulnerability.

As SCI is a physical disability, the impact on patients’ mental state tends to be ignored. Some participants believed in the occurrence of pressure sores only after they had experienced one. Attitude and beliefs can influence the prevention style adopted whether to be proactive or delegate prevention care (King et al. 2008; Zanini et al. 2020). The study by Zanini et al. (2020) found that patients with SCI, who believed they were at risk of developing pressure sores and that they could do something to prevent them from developing, were more proactive in preventing their occurrence. According to the health belief model, beliefs play an important role in health behaviour choices (King et al. 2008). Whether or not an individual adopts a specific behaviour will depend on their perception of risk to their health, the severity of the condition, their self-efficacy and the perceived benefit of the change in behaviour. To ensure holistic patient care and adoption of prevention-related behaviour, beliefs, attitudes and feelings towards SCI must be assessed during therapy.

Cognitive and behavioural therapy-based interventions could then be utilised to address patients’ beliefs, expectations and the physical components of prevention strategies (Budh, Kowalski & Lundeberg 2006; Ehde, Dillworth & Turner 2014). The use of collaborative care practice is an important strategy, where the inter-professional team, including a psychologist, can plan, implement and evaluate patient care to ensure it is holistic.

Some participants reported a lack of knowledge of SHCs and prevention care. If patients with SCI are not educated on secondary complications that could develop throughout their journey of care they will not be able to prevent their occurrence. Similar findings have been highlighted in a study based in Ghana on experiences of people with SCI (Fuseini et al. 2018) that showed significant need for more information (Van Loo et al. 2010). Lack of knowledge on SHCs is a concern because education on SHCs is fundamental to the prevention of diseases and a key focus area in achieving Sustainable Developmental Goal 3 through strengthening health literacy (World Health Organization 2017). Although knowledge on SHC does not necessarily mean positive behaviour change (Evardone et al. 2018), efforts to educate patients on health and secondary complications throughout their journey are essential. On the contrary, patients can forget what they were taught.

The forgetfulness could be because of their poor mental health state, substance abuse or the fact that it is a new behaviour that needs to be reinforced with external cues. So, it is important for health professionals to look into the principles of learning such as learning styles, applying adult learning principles, readiness to learn, and how information is taught and accessed (Manns & May 2007). The application of these teaching and learning principles has the potential to enhance knowledge levels on SHCs and prevention care.

Despite patients receiving education on SHCs, some participants were not practising prevention care. Compliance with preventive care for pressure sores amongst individuals with SCI depends on the individual’s prevention style (Zanini et al. 2020). Some of the characteristics of an individual with a compliant prevention style include extensive knowledge on SHCs, high perception of susceptibility to pressure sores, being proactive to seek support from a health professional when in need, taking responsibility for personal well-being and prioritising prevention of SHCs (Zanini et al. 2020). Individuals who are non-compliant adopt passive prevention measures, have a low perception of susceptibility and delegate responsibility for personal well-being and prevention to caregivers (Zanini et al. 2020). Part of rehabilitation for a person with a chronic condition such as SCI is to encourage patient engagement in personal health through the adoption of health-promoting behaviour.

To ensure prevention of SHCs, models such as the health belief model described above, or the stages of change can be used to encourage the adoption of new behaviour and long-term compliance.

According to the health belief model, compliance or adoption of behaviour such as prevention of SHCs depends on the individual perception of susceptibility, the severity of the health impact and barriers (King et al. 2008). It is important that during inpatient rehabilitation, education that is given on SHCs helps the patients to understand how vulnerable they are to SHCs, the seriousness of the effects of the condition and benefits of recommended preventive care practice, incorporating intrinsic (personal life goals) and extrinsic motivators (caregivers, peers) to help adopt prevention care lifestyle.

Behaviour patterns that include positive health-promoting lifestyle are good for general well-being and prevention of SHCs. Healthy lifestyle behaviours, such as exercising, not smoking or consuming alcohol, enhance prevention of SHCs. This is similar to findings in other studies that protective behaviour such as physical activity prevents or manages SHCs such as pain and depression (Tawashy et al. 2011; Zemper et al. 2003). Unhealthy behaviour such as physical inactivity, smoking and drinking alcohol lead to unhealthy outcomes and are risk factors to other SHCs such as pressure sores (Li, Dipiro & Krause 2017; Saunders & Krause 2010) and non-communicable diseases. Encouraging the adoption of healthy behaviour and increasing personal responsibility towards well-being should be part of the rehabilitation process and the primary focus of care.

Therapists could incorporate the behaviour change models described above when developing interventions or consider the Health Action Process Approach (HAPA). The Health Action Process Approach seeks to take an individual through a process of initiating, implementing and maintenance of change using constructs of setting goals, planning self-efficacy and taking action (Ma et al. 2020). Such interventions have the potential to empower patients and raise awareness of factors that influence behaviour and guide targeted interventions.

The role of a patient with chronic diseases, such as SCI, is key to chronic disease management and better health outcomes. One of the factors that emerged was patient activation, through self-management, resilience, problem-solving and help-seeking behaviour (Greene & Hibbard 2012). Patient activation is a combination of knowledge, skills, confidence and actions taken to manage health, engage healthcare and improve personal well-being (Hibbard & Greene 2013). Patients with high activation levels tend to engage more in preventive care, adopt healthy lifestyles and have better health outcomes (Greene & Hibbard 2012). To help individuals with SCI, who are vulnerable to SHCs, we need interventions that will increase awareness about their condition, skills and confidence to self-manage, promote personal health and use preventative care services. Such interventions have the potential to empower patients to take an active role in their well-being.

It is important to own appropriate assistive devices as part of preventive care. Assistive devices are essential to facilitate function, participate in life and support self-management (Bloemen et al. 2015; Burns & O’Connell 2012; Oderud 2014). In our context, the main challenge is access to basic assistive devices in terms of availability at a healthcare facility, appropriateness and the cost of purchasing or repairing assistive devices such as wheelchairs (Hanass-Hancock et al. 2017; Sherry 2015). Although universal access to assistive devices is a human right articulated in the Convention on the Rights of Persons with Disabilities, Articles 4, 20 and 26 (United Nations 2006), and is highlighted as a facilitator to achieving the sustainable development goals (Tebbutt et al. 2016), access to good quality assistive devices remains a challenge. Rehabilitation managers and clinicians can advocate for access to essential and appropriate resources as stipulated in the framework and strategy for disability and rehabilitation services in South Africa (Department of Health 2015). But to advocate for these needed resources, data collection on disability and related health outcomes must be strengthened to inform rehabilitation and primary healthcare planning, budgeting and resource allocation for needed aids.

We identified a link between the different reported personal factors. For example, if an individual with SCI does not believe they will develop SHCs they will not comply with prevention care. The relationship between the influencing factors points to the complex nature of health and the intrinsic determinants of health.

For holistic patient-centred care, rehabilitation and prevention care interventions must be tailored based on the understanding of the patients’ personal factors.

The impact of genetics and factors at a molecular level that could influence the prevention of SHCs such as UTI were beyond the scope of our study. Future research can explore factors influencing the prevention of SHCs that go beyond the psychosocial and behavioural but that include the influences at an environmental, genetic and a molecular level. In future, researchers could also consider identifying environmental factors that influence the prevention of SHCs in people with SCI. This will expand our understanding of contextual factors in the home, community and organisations that can enhance or hamper the prevention of SHCs.

## Limitations

Given that our study used a qualitative methodology, and it was conducted at a single rehabilitation facility, we cannot generalise the findings to the whole SCI population. Our study identified personal factors influencing the prevention of SHCs but did not attempt to rank them based on their level of importance.

## Conclusion

To reach the sustainable development goal number 3, patient-oriented care and preventive care for people with a chronic condition such as SCI need to be strengthened throughout the levels of care.

Understanding personal factors and addressing the barriers to the prevention of SHCs can enhance a patient-oriented care approach and inform tailored prevention strategies. To ensure effectiveness in patient care, interventions must incorporate behaviour change models, cognitive behaviour therapy and collaborative patient care practice.
